# Incidental Ingestion of Plant‐Dwelling Arthropods by Sheep and Cattle in the Same Habitat

**DOI:** 10.1002/ece3.71681

**Published:** 2025-07-02

**Authors:** Roi Forman, Maya Lalzar, Zhiwei Zhong, Deli Wang, Moshe Inbar, Tali S. Berman

**Affiliations:** ^1^ Department of Evolutionary and Environmental Biology University of Haifa Haifa Israel; ^2^ Bioinformatics Services Unit University of Haifa Haifa Israel; ^3^ Key Laboratory of Vegetation Ecology, School of Biological Sciences/Institute of Grassland Science/Jilin Songnen Grassland Ecosystem National Observation and Research Station Northeast Normal University Changchun People's Republic of China; ^4^ Key Laboratory of Grassland Resources (Inner Mongolia Agricultural University) Ministry of Education Hohhot People's Republic of China; ^5^ Department of Animal Sciences Hula Research Centre, Tel‐Hai Academic College Kiryat Shmona Israel; ^6^ MIGAL‐ Galilee Research Institute Kiryat Shmona Israel

**Keywords:** DNA metabarcoding, food webs, grazing, insects, large mammalian herbivores, trophic interactions

## Abstract

Large mammalian herbivores influence grassland ecosystems through plant consumption, return of excreta and trampling, and by altering nutrient cycles and soil properties. These herbivore‐mediated changes impact other animals in the habitat, particularly plant‐dwelling arthropods. While plant‐mediated effects of large mammalian herbivores on arthropod populations are well documented, direct effects, such as incidental ingestion of arthropods, remain understudied. Large mammalian herbivores incidentally ingest a variety of plant‐dwelling arthropods; however, it remains unclear how this interaction is influenced by the dietary choices of different mammal species within the same habitat. Using a DNA metabarcoding analysis of fecal samples, we investigated the ingestion of plant‐dwelling arthropods by sheep and cattle grazing in northeast Asian grasslands. Fecal samples were collected from replicated plots throughout the grazing season, including previously grazed and ungrazed plots. A COI marker was used to amplify arthropod DNA, followed by high‐throughput sequencing. Results revealed that both sheep and cattle ingest a variety of plant‐dwelling arthropods, including herbivores, parasitoids, and predators, with a high proportion of endophages (species developing in plant tissue). Significant differences were observed in ingestion between sheep and cattle—sheep (selective grazers) ingested a wider variety of plant‐dwelling arthropods compared to cattle, whose diet consisted primarily of grasses, highlighting the impact of dietary choices on arthropod ingestion. Grazing regime influenced the ingestion of plant‐dwelling arthropods in sheep, with differences observed between previously grazed and ungrazed plots. Our findings demonstrate that the ingestion of plant‐dwelling arthropods by large mammalian herbivores is a dynamic and widespread phenomenon, varying across mammalian species and seasons.

## Graphical abstract

In this study, a DNA metabarcoding analysis of fecal samples reveals the frequent incidental ingestion of plant‐dwelling arthropods by sheep and cattle foraging in the same habitat. Sheep and cattle ingest different varieties of arthropods. The incidental ingestion of arthropods by these large herbivores reflects their distinct dietary preferences.

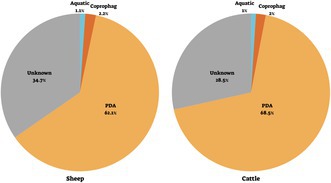

## 1 Introduction

Food webs describe trophic interactions within a community, providing a framework for ecologists to analyze energy flow, nutrient transfer, and population dynamics (Pringle and Hutchinson 2020). Grasslands, the second‐largest vegetative cover on Earth (Shui 2023), are ecosystems where large mammalian herbivores (LMH) play a crucial role—they maintain biodiversity, regulate water cycles and soil erosion, and facilitate carbon sequestration (Kang et al. 2007). They influence the diversity of many animals and plants and play essential roles in nutrient cycling and energy flow (Kang et al. 2007; van Klink et al. 2015; Zhu et al. 2015, 2024; Hassan et al. 2021; Petermann and Buzhdygan 2021; Shui 2023).

Most LMH are polyphagous, consuming diverse plant material. In doing so, they may alter vegetation structure and plant species composition (Zhu et al. 2012), indirectly affecting other organisms reliant on these plants, such as plant‐dwelling arthropods (PDA). While these plant‐mediated interactions between LMH and PDA are well‐documented (van Klink et al. 2015; Cecil et al. 2019; Zhu et al. 2023), direct interactions, such as trampling and incidental ingestion of PDA by LMH, remain largely underexplored (Gish et al. 2017).

Recent studies have highlighted the frequent incidental ingestion of various PDA by free‐ranging LMH during grazing (Berman and Inbar 2022, 2023). This frequent ingestion of PDA has likely driven the development of avoidance strategies in both organisms (Gish et al. 2017). PDA employ various escape mechanisms, such as flying, jumping, walking away, or falling off the plant when approached by feeding LMH (Gish et al. 2010; Ben‐Ari et al. 2019; Zhu et al. 2024), inducing repulsive odors (Rostás et al. 2013) or inhabiting plant parts less accessible to LMH (Bennett et al. 2015). Conversely, LMH can actively avoid consuming unpalatable or noxious PDA, which may harm them, through sensory and behavioral adaptations (Berman et al. 2017, 2018; Berman, Messeri, et al. 2019; Berman, Glasser, and Inbar 2019).

DNA metabarcoding is a non‐invasive tool used to study diet preferences and trophic interactions. It includes identifying multiple species from mixed biological samples (e.g., feces, gut contents, pollen loads, etc.) by amplifying and sequencing a short, taxonomically diagnostic genomic region using high‐throughput sequencing platforms (Clare 2014; Deiner et al. 2017; Pringle and Hutchinson 2020). In LMH, this method has been successfully used to detect diet preferences and niche partitioning in wild and domesticated species (Kartzinel et al. 2015; Garnick et al. 2018; ter Schure et al. 2021). Furthermore, this approach has been employed to study PDA ingestion by LMH, enabling the detection of functional groups that are vulnerable to ingestion, including parasitoid larvae (which are likely ingested with their hosts, i.e., secondary ingestion) and endophagous PDA (arthropods living within plant tissues) that are unable to escape the plant (Berman and Inbar 2022, 2023). While recent research has assessed the extent and frequency of PDA ingestion by LMH, how these trophic interactions may vary in the same habitat among different LMH species with unique feeding preferences remains unknown.

In this study, we used a DNA metabarcoding analysis of LMH feces to identify and compare the PDA ingested by sheep and cattle grazing in the same habitat, the grasslands of northeast China. Previous studies in the area demonstrated that sheep and cattle grazing in this system exhibit different foraging strategies, with each selecting different plant species (Forman et al., in press). Sheep were found to be more selective than cattle (Liu et al. 2015; Zhu et al. 2017), typically avoiding grasses (Poaceae), the dominant plant group in the area. Instead, they preferred forbs (non‐grass perennials, such as Fabaceae and Asteraceae), particularly the dominant forbs Artemisia, which are higher in nutrients than grasses (Li et al. 2021; Zhong et al. 2014). In contrast, cattle were less selective, consuming the dominant plants, primarily the dominant grasses *L. chinensis* and *Phragmites australis* (Li et al. 2023; Zhong et al. 2022). Accordingly, we addressed the following questions: (1) Which functional and taxonomic groups of PDA are ingested by sheep and cattle while grazing? (2) Does the composition of PDA communities ingested by cattle and sheep vary throughout the grazing season? and (3) Does the composition of PDA communities ingested by cattle and sheep differ between sites previously exposed to grazing and sites that were not grazed? We hypothesized that sheep and cattle would ingest different arthropods while grazing due to their distinct dietary preferences (Grant et al. 1985; Liu et al. 2015; Zhu et al. 2015).

## 2 Materials and Methods

### 2.1 The Study System

The study was conducted at the Grassland Ecological Research Station of Northeast Normal University, Jilin Province, China (44°45′ N, 123°47′ E). It is located in a meadow steppe region, a semi‐arid grassland with annual precipitation averaging 280–400 mm (primarily between May and October) (Zhu et al. 2012). The plant community mainly consists of perennial grass species (family Poaceae), with *Leymus chinensis* being the dominant species in the area, and some forb species such as *Kalimeris integrifolia* and *Artemisia scoparia* (Zhu et al. 2012, 2017), as well as legume species like *Lespedeza davurica*. Previous studies found a large variety of PDA in the area from 57 families and 9 orders, with PDA from the orders Orthoptera and Hemiptera being particularly common (Zhu et al. 2012).

Livestock grazing is the main land‐use practice in these natural grasslands. Grazing takes place annually from May to October during the growing season, with house‐feeding occurring during the rest of the year. Cattle and sheep are the primary large grazers, providing meat and milk for the local community. Furthermore, cattle and sheep owned by local farmers are often used for controlled grazing experiments in this area, which occur only during the hot and humid summer due to the harsh, frozen winter.

### 2.2 Experimental Design

**FIGURE 1:**
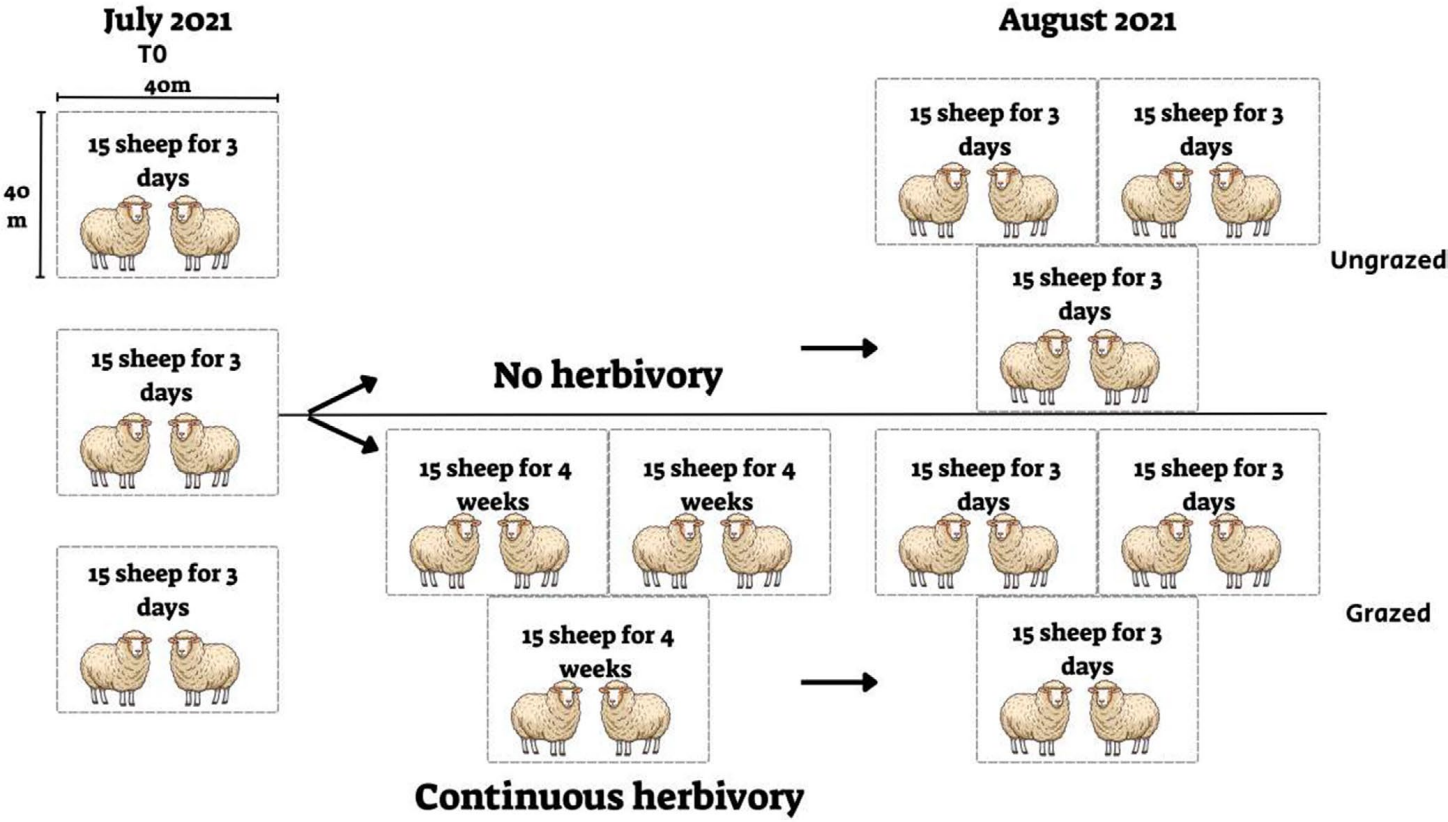
Sheep experimental design. On July 2021 (T0), three 40 m × 40 m plots were set up. Fifteen sheep were assigned to each plot for 3 days. On August 2021, two new replicated sets were set up, three plots in an area without previous disturbances (ungrazed) and three plots in a sheep‐grazed area (grazed). See text for the slightly modified experimental design of cattle grazing.

In July 2021, three fenced 40 m × 40 m plots were randomly set up in an area with no disturbances (grazing or mowing). The distances between the plots were at least 10 m (Figure 1). Fifteen sheep (randomly selected from a single herd) were assigned to each plot for three days. At the end of the third day, the grazing ceased, and 15 random fecal samples, containing 10–15 fresh fecal pellets each, were collected. The samples were at least 5 m apart to prevent pseudo‐replication (collecting from the same individual twice). This sample collection time is referred to as “T0”. One month later, in August 2021, we established two new sets of three fenced 40 m × 40 m plots – one set in an area that had remained ungrazed during the previous month (“ungrazed”), and another set in an area that had been continuously grazed by 15 sheep per plot for four weeks (“grazed”). As described above, 15 sheep were assigned to each plot in these sets for three days, and their feces were collected (plot design, grazing protocols, and fecal sample collection were identical to those described for the “T0” plots above). This setup enabled us to examine the impact of recent grazing (compared to non‐recent grazing) on the PDA community detected in LMH feces. Each fecal sample was placed in a plastic tube and kept at −80°C until processing. In total, 135 fecal samples of sheep were collected in July and August 2021 (45 samples for T_0_ and 90 samples for grazed and ungrazed plots).

In the summer of 2022, a similar experimental design was set up for cattle. The size of the plots and the duration of grazing were the same as described above (Figure 1). Due to the low availability of cows from the local farmers, during the continuous grazing phase (of 4 weeks), only 3–4 cows were used per plot. During sampling times (T0, grazed, and ungrazed), 15 cows were assigned to each plot. Fecal samples were collected with a spatula from several parts within the center of the dung pile and were handled according to the same protocols used in the sheep experiment. Further details regarding the samples, treatments, and study site are provided in Dataset S1.

### 2.3 DNA Extraction and Amplification

Prior to DNA extraction, each fecal sample was thoroughly mixed and homogenized to ensure uniform and consistent samples (i.e., ensure even distribution of the fecal matter, preventing potential biases during DNA extraction). DNA extraction was performed using the QIAamp Fast DNA Stool Mini Kit (50 preps., Cat. No. 51604, QIAGEN, Hilden, Germany), using the manufacturer protocol (in step 3, lysis temperature was increased to 95°C and in step 8, incubation time was increased to 20 min). The amplification of arthropod DNA was performed using the Zeale et al. (2011) COI mitochondrial markers (~157 bp amplicon). These markers were chosen because they are supported by a large reference database and were already found useful in family‐level discrimination of PDA (Berman and Inbar 2022, 2023). PCR conditions were 98°C for 2 min, followed by 35 cycles of 90°C for 10 s, 52°C for 30 s, and 72°C for 30 s. PCR was completed with 1 min of 72°C. Negative controls were carried out for each PCR assay. We verified the amplification by agarose gel electrophoresis and stored the PCR products at −20°C.

### 2.4 Sample Sequencing and Library Preparation

PCR products, as well as negative controls, were sequenced on an Illumina MiSeq system (Illumina, San Diego, CA, USA) at the Genomics and Microbiome Core Facility (GMCF), Rush University, Chicago, USA. Briefly, A second PCR amplification was performed in 10 μL reactions in 96‐well plates using repliQa HiFi ToughMix (2×mastermix; 2uL of the primer pair was added to the reaction on top of the repliQa HiFi ToughMiX—QuantaBio). Each well received a separate primer pair with a unique 10‐base barcode, obtained from the Access Array Barcode Library for Illumina (Fluidigm, South San Francisco, CA; Item# 100‐4876). One microliter of PCR product from the first stage amplification was used as a template for the 2nd stage without cleanup. Cycling conditions were 98°C for 2 min, followed by 8 cycles of 98°C for 10 s, 60°C for 1 min and 68°C for 1 min. Libraries were then pooled and sequenced with a 15% phiX spike‐in on an Illumina MiSeq sequencer employing V3 chemistry (2 × 300 paired‐end reads). Sequencing depth was a mean of 87,607 and 105,208 reads per sample for sheep and cattle, respectively.

### 2.5 Sequence Analysis and Bioinformatics

Bioinformatic analysis was performed at the Rush Research Bioinformatics Core, Rush University Medical Center, Chicago, USA, using QIIME2 2021.11 (Bolyen et al. 2019). Raw sequence (FASTQ files) data was checked for quality by using FastQC and merged using PEAR default parameters. Merged sequences were imported into the QIIME2 environment using the qiime tools import algorithm. Manifest and metadata files were created accordingly and used for the import. Primer sequences were removed using the qiime cutadapt trim‐single algorithm. Anything less than 100 bp was removed. Reads that did not have both primer sequences were also removed. The qiime dada2 denoise‐single algorithm (Callahan et al. 2016) was used to denoise the reads. No length‐based truncation was set in dada2 denoise‐single (–p‐trunc‐len 0). Abundance tables containing amplicon sequence variants (ASVs) were generated using the biom convert function. ASVs found in sheep and cattle samples were arranged in two separate tables containing 12,145,641 reads binned in 2477 ASVs for sheep and 10,864,732 reads binned in 2298 ASVs for cattle. ASV sequences were aligned against the NCBI GenBank nt‐database. BLAST output files were imported into ‘MEGAN community edition’ (v.6.21.11; (Huson et al. 2016)) for taxonomic classification. The taxonomic classification was performed using the Lowest Common Ancestor (LCA) approach with the following parameters: bitscore > 100 (to consider only high‐confidence matches), E value < 10^7^ (to limit random matches and include only meaningful hits), min support = 1 (to ensure rare taxa are included), top percent = 10 (to focus the analysis on the top 10% of the best matches), LCA algorithm = naïve, and percent to cover = 100 (to ensure the entire query sequence is aligned to the database).

### 2.6 Data Analysis and Statistics

Data analysis was performed separately for sheep and cattle.

We initially identified 2477 ASVs in sheep samples and 2298 ASVs in cattle samples. We removed any ASVs found in negative controls, resulting in 2458 ASVs in sheep samples and 2292 ASVs in cattle samples. Next, we removed all ASVs that did not receive any taxonomical assignment (327 ASVs in sheep and 282 ASVs in cattle), and ASVs that were not assigned to the phylum Arthropoda (mostly Nematodes and Rotiferas; 103 ASVs in sheep and 89 ASVs in cattle, Dataset S2). To ensure a more precise assessment of the arthropod community, ASVs appearing in only one sample were excluded (Dataset S3). This process resulted in 10,953,341 reads across 429 ASVs for sheep and 8,154,182 reads across 323 ASVs for cattle (Dataset S4).

Drawing upon insights from the existing literature, all arthropod taxa were assigned to functional feeding guilds, including herbivores, predators, parasitoids, dung‐associated arthropods, and aquatic arthropods. Herbivorous arthropods were further classified by their feeding niche: endophages (those that feed within plant tissues, such as leaf miners and gall‐forming arthropods) or exophages (those that feed on external plant surfaces). Taxa lacking sufficient information for classification were designated as unknown. This was done using the species level when available, followed by the order, and in some cases, the family.

For a PDA analysis (i.e., only plant‐dwelling arthropods, excluding dung‐associated and aquatic arthropods), a subset of the data was created, including only ASVs assigned to herbivores, predators, and parasitoids, retaining 259 ASVs for sheep (6,551,961 reads) and 212 ASVs for cattle (5,553,903 reads; Dataset S5). For all four datasets (two each for sheep and cattle), the data were rarefied to 10,000 reads per sample (Appendix S1). In the “all arthropods” datasets, 134 sheep and 124 cattle samples were retained (Dataset S6). In the PDA datasets, 133 sheep samples and 119 cattle samples were retained (Dataset S7).

Diversity analysis was performed both by calculating the relative read abundance (RRA), which is the proportion of reads of each ASV in each fecal sample, and by calculating the proportion of occurrence of taxa (POO, calculated as the number of samples containing a given food item, rescaled to 100% across all food items, see (Deagle et al. 2018)). We chose RRA as the primary basis for inference in this study. We used a supporting analysis of presence/absence as a sensitivity check against the possible biases in of RRA, such as different digestibility of PDA by LMH and amplification biases (Appendix S2). RRA and POO were calculated separately for all arthropod ASVs and ASVs of PDA alone. In both RRA (Datasets S8 and S9) and POO (Datasets S10 and S11), a “true presence” threshold was set—ASVs with an abundance of < 1% within a sample were removed (i.e., true presence threshold, see (Deagle et al. 2018)).

Shannon diversity (H′) and Fisher's alpha (α) indices were calculated using the “vegan” R package to assess the diversity of PDA communities ingested by sheep and cattle. Differences between treatments (grazed vs. ungrazed) for these indices were analyzed using a Kruskal–Wallis test (using the kruskal.test function in the R “stats” package). We used the Kruskal–Wallis test to compare how the proportions of different PDA feeding guilds (herbivores, parasitoids, and predators) and herbivorous PDA feeding niches (endophages, exophages, and unknown) varied across the treatments. Dunn's post hoc test with Bonferroni correction for multiple comparisons was then performed using the “dunn.test” R package.

Next, we performed cumulative‐sum scaling (CSS) normalization on each PDA subset (pre‐subsampling data), to account for variation in sequencing depth across samples (Paulson et al. 2013) using the “cumNorm” function in R “metagenomeSeq” package. To investigate the similarity of PDA communities between treatments, a non‐metric multidimensional scaling (NMDS) analysis was performed for sheep and cattle separately using R “vegan” function “metaMDS” (Bray–Curtis similarity matrix). To test whether the PDA communities differed between treatments (T0, grazed and ungrazed), a permutational multivariate analysis of variance (PERMANOVA) was conducted, with 999 permutations (distance measure: Bray‐Curtis).

## 3 Results

In total, we identified 262 ASVs of arthropods in sheep samples and 267 ASVs in cattle samples (RRA of > 1%), after filtering out non‐arthropod and low‐abundance ASVs. The ASVs identified in sheep represented 59 species, 63 genera, and 47 families in eight orders of insects (96% of RRA, 95% of POO) and 10 species, nine genera, and eight families in two orders of arachnids (4% of RRA, 5% of POO). In cattle, the identified ASVs represented 76 species, 83 genera, and 49 families in seven orders of insects (97% of RRA, 96% of POO) and 11 species, eight genera, and eight families in two orders of arachnids (3% of RRA, 4% of POO).

**FIGURE 2:**
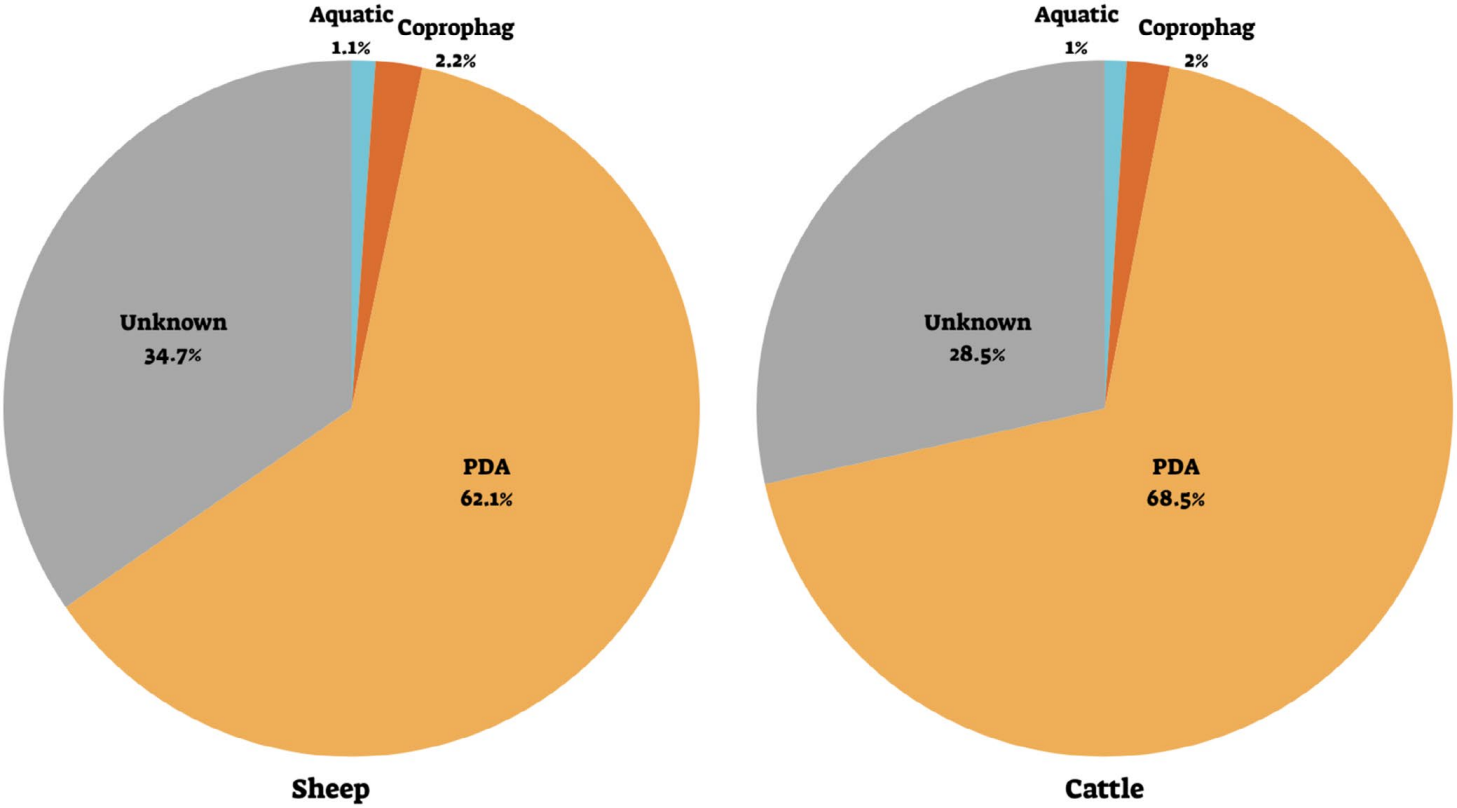
The guilds of arthropods identified in sheep (left) and cattle (right) feces. Values are the mean proportion of reads (RRA) averaged across all three treatments (T0‐ beginning of the season with no previous grazing, grazed—previously grazed, ungrazed‐ no previous grazing).

The ASVs were assigned to functional groups and guilds. PDA were the main group, comprising over 60% of sequences in sheep and cattle samples (both of RRA and POO; Figure 2). Additionally, the proportions of dung‐associated (coprophagous) arthropods, which may have contaminated the fecal samples during collection, and aquatic arthropods (likely ingested while drinking), were similarly low in both the sheep and cattle feces (in both RRA and POO; Figure 2). About 30% of the RRA (and of POO) were ASVs who's functional group could not be determined and remained unknown (Figure 2).

### 3.1 PDA Ingested by Sheep and Cattle

**FIGURE 3:**
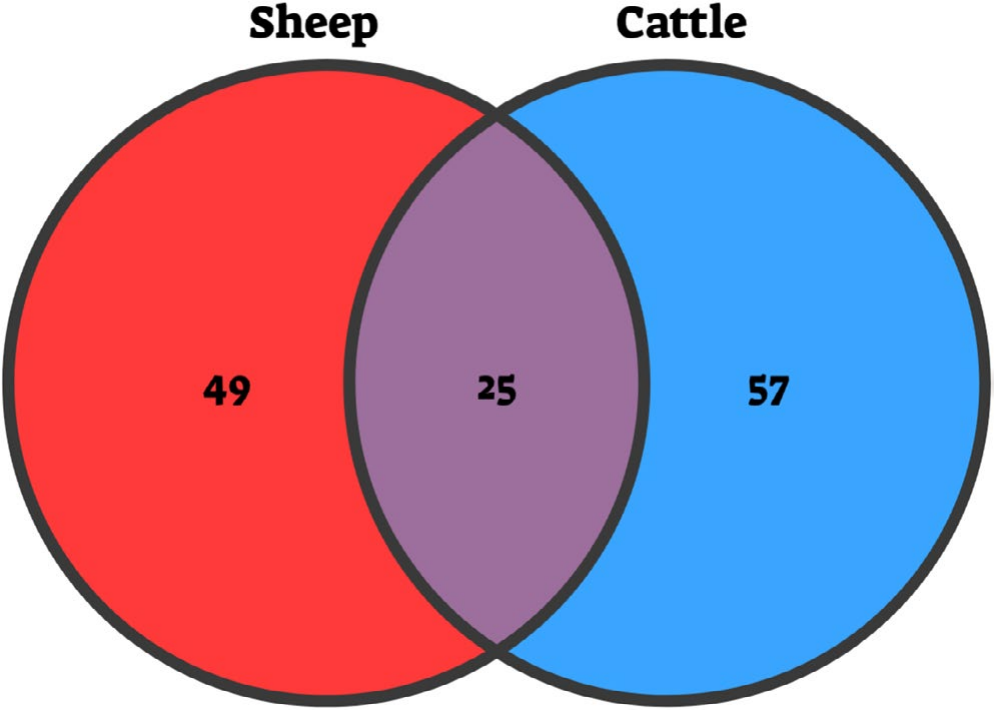
Distribution of plant‐dwelling arthropod (PDA) genera ingested by sheep and cattle. The diagram shows the number of PDA genera identified in fecal samples from sheep (left) and cattle (right), and genera shared between both herbivores (center).

We identified a complex food web of PDA ingested by sheep and cattle, including herbivores, predators, and parasitoids. The frequency of PDA ingestion by the two LMH was high—DNA assigned to PDA was detected in 133 of 135 sheep samples (98%) and 119 of 135 cattle samples (88%). In both sheep and cattle, we identified 131 genera of PDA, with a 20% overlap of genera. This overlap included *Aphis* (Hemiptera), *Coleophora* (Lepidoptera), and *Negayan* (Araneae; Figure 3; A full list of overlapping genera is provided in Dataset S12). At the family level, Cecidomyiidae (Diptera) was the most abundant PDA ingested by both LMH, comprising 34.5% of RRA and 23.4% of POO in sheep, and 25.9% of RRA and 25.0% of POO in cattle. The Aphididae (Hemiptera) family was also common, with 9.4% of RRA and 5.0% of POO in sheep, and 4.6% of RRA and 7.1% of POO in cattle. Taking into account that a large portion of ASVs could not be identified to the genus level (86.3% of RRA and 65.8% of POO in sheep, and 57.6% of RRA and 52.9% of POO in cattle), the most ingested genera that were identified included *Aphis* (Hemiptera) in both sheep (8.2% of RRA, 3.6% of POO) and cattle (1.8% of RRA, 2.6% of POO). In sheep, *Anarta* (Lepidoptera; 3.8% of RRA, 1.8% of POO) and *Voria* (Diptera; 3.1% of RRA and of POO) were also abundant. In cattle, *Agrius* (Lepidoptera; 21.1% of RRA, 7.2% of POO) and *Galerucella* (Coleoptera; 3.3% of RRA, 3.8% of POO) were among the most dominant genera.

**FIGURE 4:**
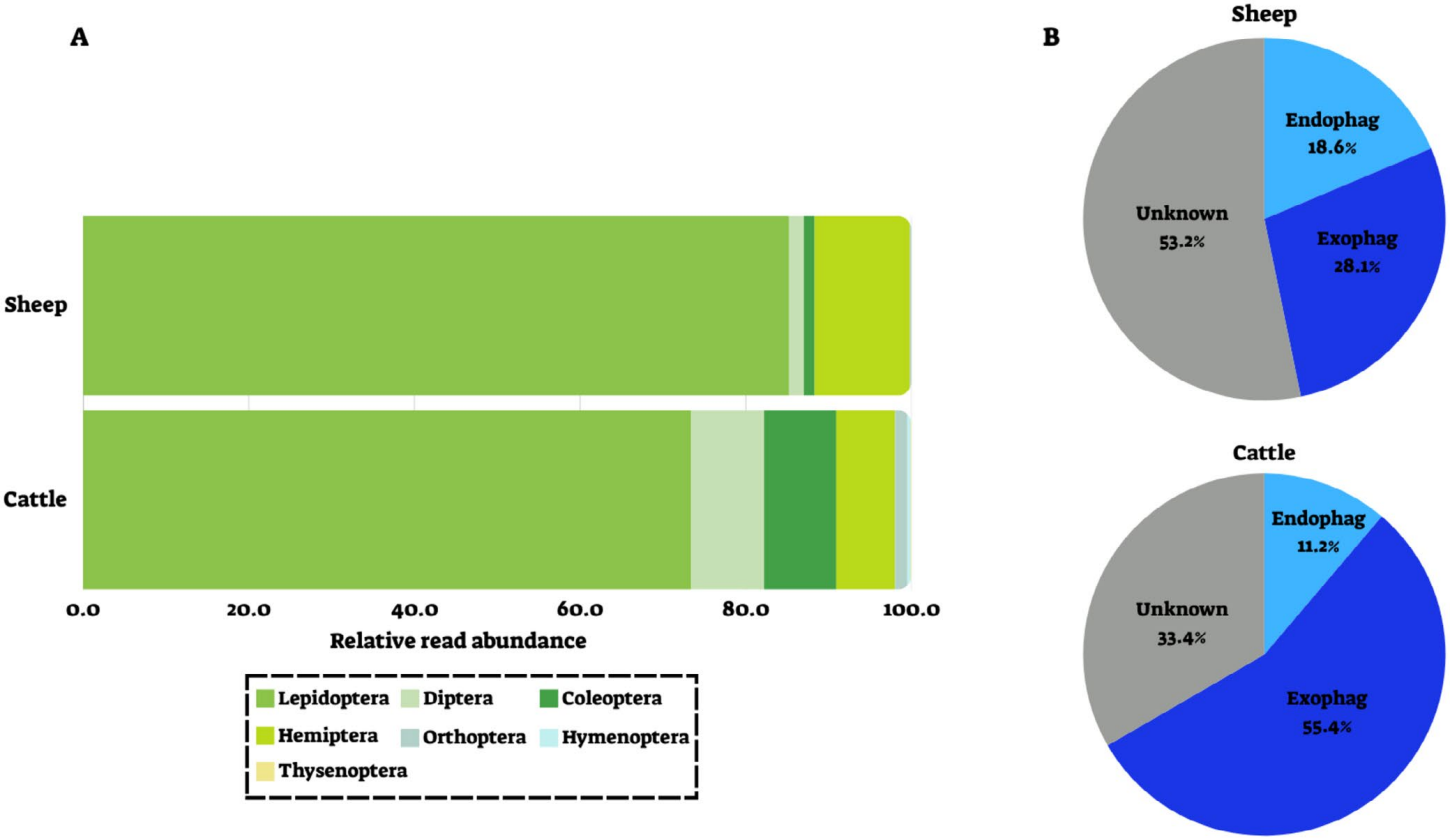
Composition of herbivorous PDA ingested by sheep and cattle. Values are the mean proportion of reads (RRA) averaged across all three treatments. (A) Proportions of herbivorous PDA orders detected in sheep and cattle fecal samples. (B) Feeding niches of ingested herbivorous PDA (exophages—feed on the plant surface; endophages—feed within plant parts).

We found 55 species of herbivorous PDA in sheep samples from 50 genera, 28 families, and five orders (88.7% of RRA, 85.7% of POO). In comparison, in the cattle samples, we found 59 species, 65 genera, and 37 families in 7 orders of herbivorous PDA (93.1% of RRA, 90.8% of POO). In both LMH, these ASVs belonged mainly to the order Lepidoptera (Figure 4A). Most of the herbivores identified in cattle samples were exophages (55.4% of RRA, 53.8% of POO, Figure 4B), while in sheep the exophagous herbivores accounted for 28.1% (RRA, 30.2% of POO, Figure 4B). Endophages comprised 18.6% of herbivorous PDA in sheep (RRA, 25% of POO) and 11.2% in cattle (RRA, 16.9% of POO).

**FIGURE 5:**
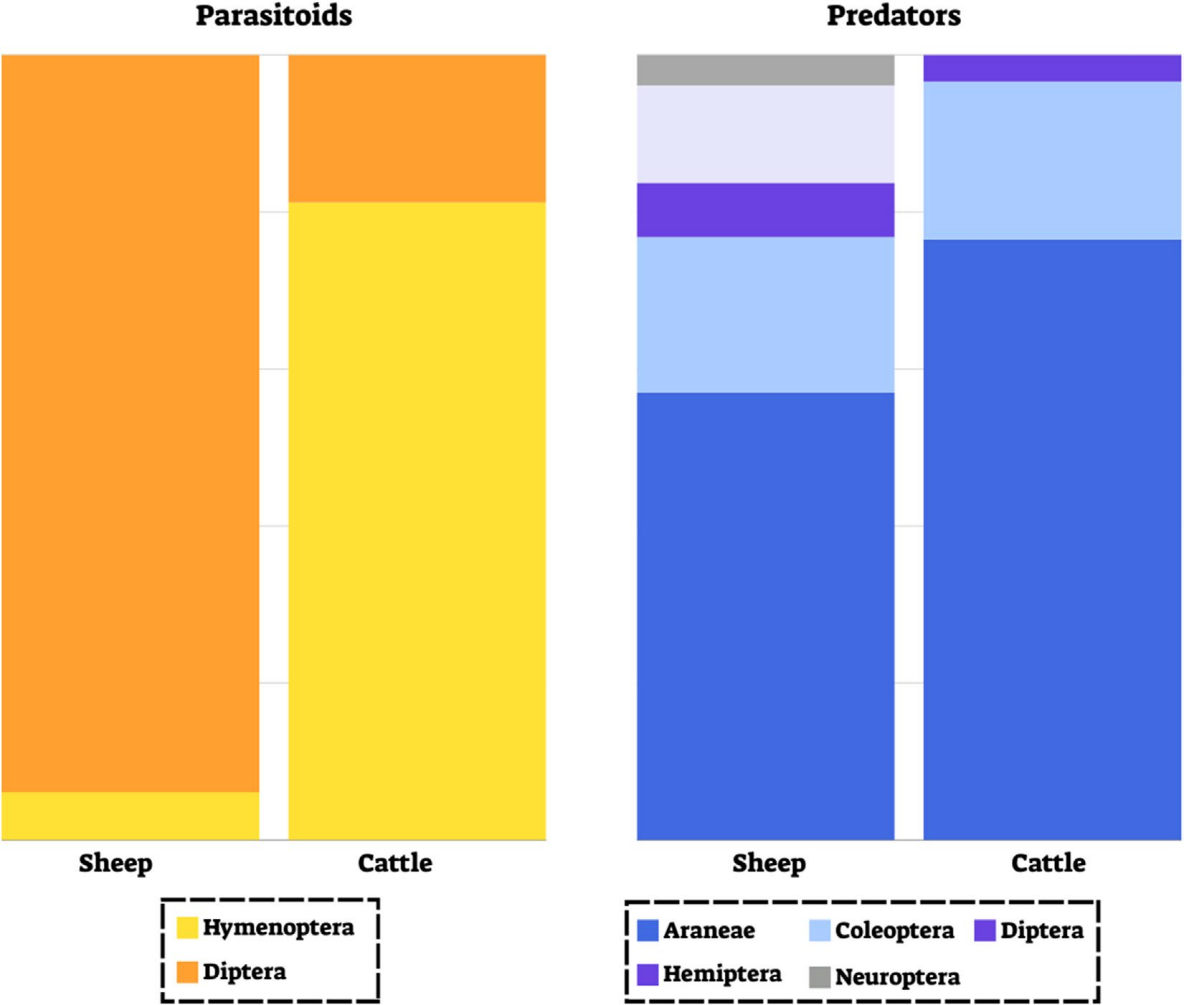
Composition of parasitoids (Left) and predatory (Right) PDA ingested by sheep and cattle. Values are the mean proportion of reads (RRA) averaged across all three treatments.

In both sheep and cattle, we found two orders of parasitoids—Hymenoptera and Diptera, which comprised less than 1% of all reads in cattle (both of RRA and POO) and 6.1% in sheep (RRA; 7.8% of POO). While in cattle, the parasitic wasps (Hymenoptera) comprised over 80% of the total parasitoids ingested, in sheep samples, we identified about 6% of this order (Figure 5).

The predators identified in sheep fecal samples belonged to 14 species, 19 genera and 16 families in five orders (5.1% of RRA, 6.1% of POO), mainly Araneae and Coleoptera (Figure 5). These orders were also abundant in cattle samples, together with a smaller proportion of Diptera (Figure 5). The predatory arthropods found in the cattle feces represented 12 families, 11 genera and 10 families (6.1% of RRA, 8.3% of POO).

### 3.2 Variation Between Treatments in PDA Ingested by Sheep and Cattle

**FIGURE 6:**
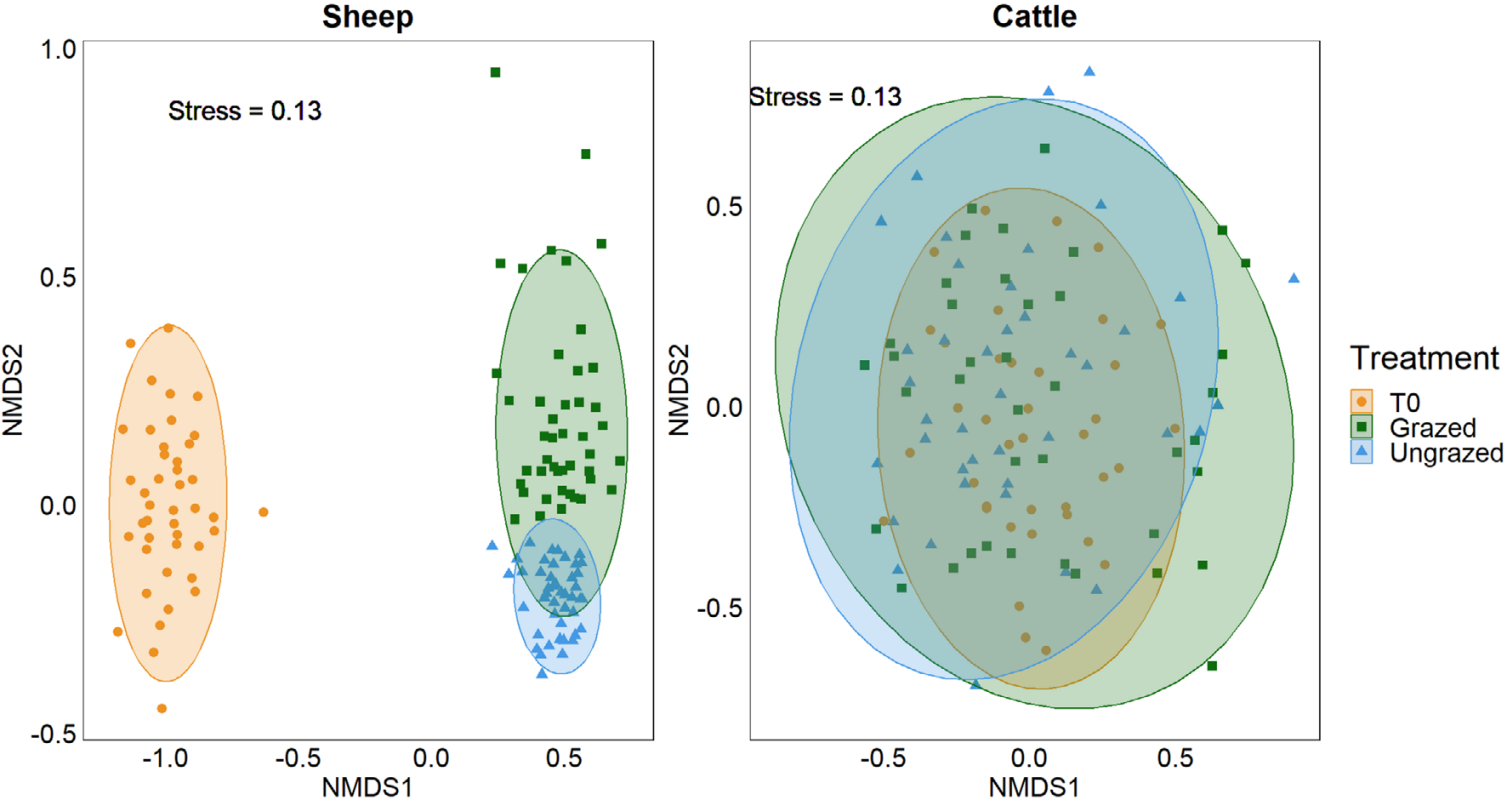
Similarity of plant‐dwelling arthropod (PDA) communities in sheep (Left) and cattle (Right) fecal samples across all three treatments (T0‐ beginning of the season with no previous grazing, grazed—previously grazed, ungrazed‐ no previous grazing). The non‐metric multidimensional scaling (NMDS) analysis is based on Bray–Curtis dissimilarity matrix with 95% confidence ellipses. Each point represents the PDA composition of a single fecal sample. Orange points: Samples from T0; Green points: Samples from grazed plots; Blue points: Samples from ungrazed plots.

We found significant changes in the composition of PDA detected in sheep samples over the three treatments (ADONIS: *R* = 0.4, *p* < 0.001), yet not in cattle samples (ADONIS: *R* = 0.02, *p* = 0.085; Figure 6). While in cattle samples we found substantial overlap between the PDA composition of all three treatments, in sheep samples we identified a major difference between samples from T0 and samples from grazed and ungrazed plots, indicating a seasonal shift in ingested PDA. We also found a clear difference in PDA composition between sheep samples collected from grazed and ungrazed plots (Figure 6).

**FIGURE 7:**
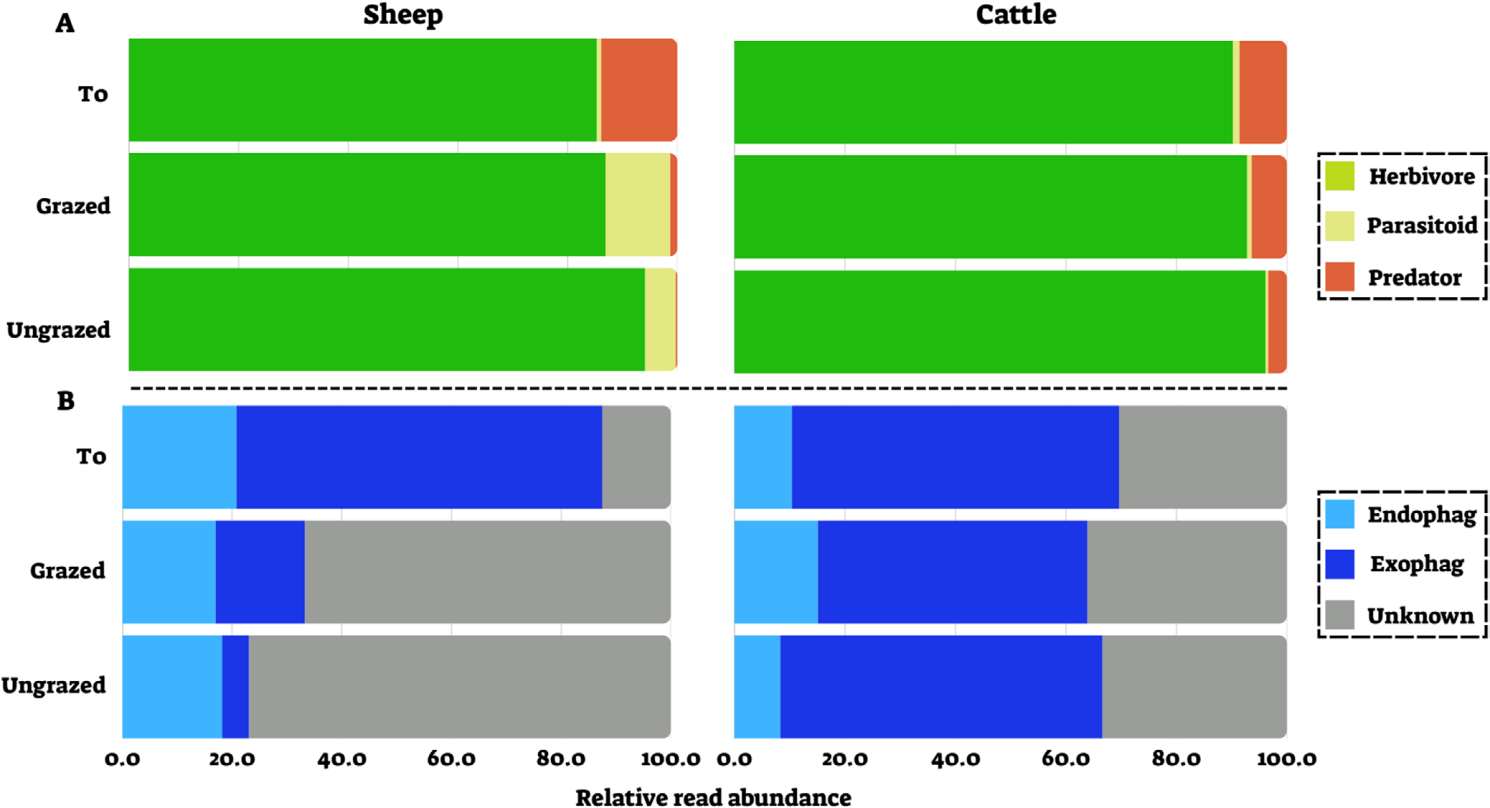
Composition of plant‐dwelling arthropods (PDA) ingested by sheep (left) and cattle (right) across the three treatments (T0‐ beginning of the season with no previous grazing, grazed—previously grazed, ungrazed‐ no previous grazing). (A) Mean relative abundance of feeding guilds (herbivores, parasitoids and predators). (B) Mean relative abundance of feeding niches of herbivorous PDA (exophages—feed on the plant surface; endophages—feed within plant parts).

In sheep, PDA from different feeding guilds (herbivores, parasitoids, and predators) and herbivorous PDA from different feeding niches (exophages and unknown) varied significantly across treatments (Figure 7, Kruskal–Wallis test, *p* < 0.05 for all significant comparisons), while endophagous herbivores showed no significant variation. PDA detected in cattle feces showed no significant variation in feeding guild or herbivore feeding niche (Figure 7, Kruskal–Wallis test, *p* > 0.1 for all comparisons; see Dataset S13 for full statistical details).

Diversity (Shannon H′) and richness (Fisher's α) also significantly differed between treatments in sheep samples, yet not in cattle samples (Table 1). In sheep samples, the diversity was higher in grazed plots, compared to T0 and ungrazed plots, while the richness was highest in ungrazed plots (Table 1).

**TABLE 1:** Diversity of plant‐dwelling arthropod communities ingested by sheep and cattle.

Treatment	Shannon H′		Fisher's α	
	Sheep	Cattle	Sheep	Cattle
T0	1.9 ± 0.05^b^	1.3 ± 0.12	2.07 ± 0.09^c^	1.4 ± 0.12
Grazed	2.19 ± 0.05^a^	1.39 ± 0.14	2.97 ± 0.13^b^	1.56 ± 0.18
Ungrazed	1.94 ± 0.03^b^	1.3 ± 0.1	3.66 ± 0.13^a^	1.51 ± 0.11

*Note:* Comparison between T0 (beginning of the season with no previous grazing), grazed (previously grazed) and ungrazed (no previous grazing) plots, different letters indicate significant difference based on Kruskal–Wallis test.

## 4 Discussion

We used a molecular analysis to identify arthropods ingested by sheep and cattle grazing in the same habitat. We found that sheep and cattle ingested various arthropods, primarily PDA, including the functional groups—herbivores, parasitoids, and predators. Among the herbivorous PDA, a significant proportion were identified as endophagous species—arthropods that feed within plant tissue (Figure 4B). This group predominantly consisted of gall midges (Cecidomyiidae), leaf miners (Agromyzidae), casebearers (Coleophoridae) and leaf borers (Gracillariidae) (from the orders Lepidoptera and Diptera). Endophagous species have previously been found to be vulnerable to LMH ingestion due to their inability to escape the plant (Berman and Inbar 2022, 2023). Yet detecting the ingestion of these PDA is nearly impossible when using traditional methods, as they are cryptic during most of their life (Schlinkert et al. 2015). Our findings highlight the advantage of using DNA metabarcoding analysis of LMH fecal samples as a tool for identifying a variety of PDA vulnerable to ingestion.

Other ingested PDA included small, wingless species like aphids (Aphididae) and possibly immature life stages of mobile arthropods, such as moths (Lepidoptera), parasitic wasps, and flies (Hymenoptera and Diptera). While distinguishing between life stages is impossible using DNA metabarcoding alone, we assume that the young, immobile stages were ingested and not the adults, who can escape ingestion. Although grasshoppers are very common in the study site (Zhu et al. 2012), they comprised only a minor component of the PDA consumed by sheep and cattle (Figure 4A). Similarly, other arthropods, such as bees and ants that are abundant in the field (Zhu et al. 2012), were absent from the fecal samples of both species. When facing the risk of being ingested by grazing LMH, highly mobile arthropods can jump or fly away (Gish et al. 2017; Zhu et al. 2024), and noxious or aggressive arthropods may deter LMH (Martins 2010; Berman et al. 2017), which could explain the low abundance of these PDA in the fecal samples. Overall, RRA and POO quantifications were generally similar (Mantel test, *p* = 0.001; see Appendix S2).

### 4.1 Seasonal and Species‐Specific Variation in PDA Ingestion

The grazing season in the grasslands of northeast Asia occurs during the summer months (July–September). Hence, perennial plants grow rapidly and reach peak growth during August (Zhong et al. 2014). We hypothesized that this seasonality would be reflected in the ingested PDA, which rely on these plants for food and shelter. We found a clear seasonal shift in the composition of the PDA community ingested by sheep, with a distinct separation between the beginning of the season (T0) and the end of the season (grazed and ungrazed; Figure 6). Surprisingly, this seasonal pattern was less pronounced in cattle (although T0 samples appeared to cluster, suggesting some level of distinctness; Figure 6). The observed differences in PDA ingestion between sheep and cattle likely stem from variations in their foraging behaviors and dietary selectiveness. Most herbivorous PDA exhibit high plant specificity (Jaenike 1990; Forister et al. 2015), which may result in a relatively small overlap in the PDA genera ingested by these LMH as they select different plants to feed from (Figure 3). Sheep are more selective grazers than cattle, favoring nutrient‐rich forbs and legumes over grasses (Grant et al. 1985; Cuchillo‐Hilario et al. 2018; Wang, Wang, et al. 2018; Li et al. 2021). In contrast, cattle tend to consume a greater quantity of food to maximize performance and consume mainly grasses (Mphinyane et al. 2015; Wang, Wang, et al. 2018; Wang, Yuan, et al. 2018). Since grasses are the most common plants in the study area, cattle diet likely does not vary significantly throughout the year (Grant et al. 1985). The dietary selectivity of sheep likely exposes them to PDA communities associated with greater plant species diversity, leading to pronounced seasonal variation in the arthropods they ingest. The greater diversity of PDA ingested by sheep than that of cattle mirrors patterns previously observed in other LMH. Goats were found to ingest a more diverse range of PDA than cattle (Berman and Inbar 2022, 2023), likely due to being more selective. These findings demonstrate the powerful role of dietary selectivity in shaping this trophic interaction.

### 4.2 Impact of Grazing on PDA Ingestion

We observed significant differences in the PDA ingested by sheep between grazed and ungrazed plots, a pattern not present in cattle. While there was some overlap in the PDA community composition between these treatments, the samples collected from ungrazed plots showed greater similarity to one another compared to those collected from grazed plots (Figure 6). This suggests that grazing alters the composition of the PDA community in grazed areas, ultimately influencing the PDA ingested by sheep in areas that have been previously grazed. Grazing can alter plant community composition by decreasing plant cover and height and changing the relative abundance of different plants (Liu et al. 2015; Zhong et al. 2017). Due to the selective grazing habits of sheep, the abundance of their preferred plant species would be reduced in previously grazed areas. This decrease in preferred plants likely results in a more varied diet, as sheep in previously grazed areas are driven to consume a broader selection of available plants, increasing the diversity of ingested PDA. Furthermore, we observed greater Shannon H' diversity in grazed plots compared to ungrazed plots, indicating that grazing might result in a more uniform distribution of ingested PDA, possibly as a consequence of this more diverse diet.

### 4.3 Ecological Significance

In this study, we demonstrate that sessile PDA and immature life stages (endophages developing in plant tissue, parasitoid larvae ingested with their host, relatively immobile stages such as eggs, pupae, and larvae, all characterized by reduced mobility) are vulnerable to being ingested by feeding LMH. This is likely to be particularly important when considering vulnerable or endangered PDA in chronically grazed systems.

The frequent ingestion of PDA by LMH may affect other animals within the shared habitat. For example, by reducing the population of PDA, LMH may considerably impact other insectivorous animals in the shared habitat (such as birds, reptiles, and other small mammals), which depend on them as a food source.

The frequent ingestion of PDA by LMH, along with the fact that both PDA and LMH developed specific adaptations to minimize the risk of ingestion (Gish et al. 2010; Ben‐Ari and Inbar 2013; Rostás et al. 2013; Bennett et al. 2015; Berman et al. 2017, 2018; Ben‐Ari et al. 2019; Berman, Glasser, and Inbar 2019), indicates that this trophic interaction is a prevalent and important part of grassland ecosystems. While the consequences for PDA are mostly lethal, LMH and herbivores in general may benefit from PDA ingestion by supplementing their plant‐based diet with protein and micronutrients (Berman and Inbar 2025).

### 4.4 Conclusion

In this study, we reveal that PDA ingestion by LMH is a dynamic and widespread phenomenon, common not only in sheep and cattle in the grasslands of northeast China, but also in other LMH, including goats (Berman and Inbar 2022, 2023) and cattle (Berman and Inbar 2022, 2023) feeding in Mediterranean habitats. Despite feeding in the same habitat, we show that sheep and cattle affect (through ingestion) different groups of PDA due to their distinct dietary preferences (Forman et al., in press). The results of this study emphasize the considerable influence of the dietary preferences of LMH on the trophic interactions between LMH and PDA.

## Author Contributions

**Roi Forman:** conceptualization (equal), data curation (lead), formal analysis (lead), writing – original draft (lead). **Maya Lalzar:** data curation (supporting), formal analysis (supporting), writing – review and editing (equal). **Zhiwei Zhong:** conceptualization (equal), methodology (lead), writing – review and editing (equal). **Deli Wang:** conceptualization (equal), funding acquisition (lead), methodology (lead), writing – review and editing (equal). **Moshe Inbar:** conceptualization (equal), data curation (supporting), formal analysis (supporting), funding acquisition (lead), methodology (lead), supervision (lead), writing – review and editing (equal). **Tali S. Berman:** conceptualization (equal), data curation (supporting), formal analysis (supporting), methodology (lead), writing – review and editing (equal).

## Conflicts of Interest

The authors declare no conflicts of interest.

## Supporting information


Appendix S1.

Appendix S2.

Dataset S1.

Dataset S2.

Dataset S3.

Dataset S4.

Dataset S5.

Dataset S6.

Dataset S7.

Dataset S8.

Dataset S9.

Dataset S10.

Dataset S11.

Dataset S12.

Dataset S13.

## Data Availability

Data available from the National Center for Biotechnology Information (NCBI) GenBank: accession number PRJNA1083309.
