# Supplementary material for: Incidental Ingestion of Plant‐Dwelling Arthropods by Sheep and Cattle in the Same Habitat
Source: Ecol Evol. 2025 Jul 2;15(7):e71681. doi: 10.1002/ece3.71681 (PMC12222620; doi:10.1002/ece3.71681)
Supplement: Supplementary file 1 — Appendix S1. [file ECE3-15-e71681-s004.docx]

**Incidental ingestion of plant-dwelling arthropods by sheep and cattle in the same habitat**

**
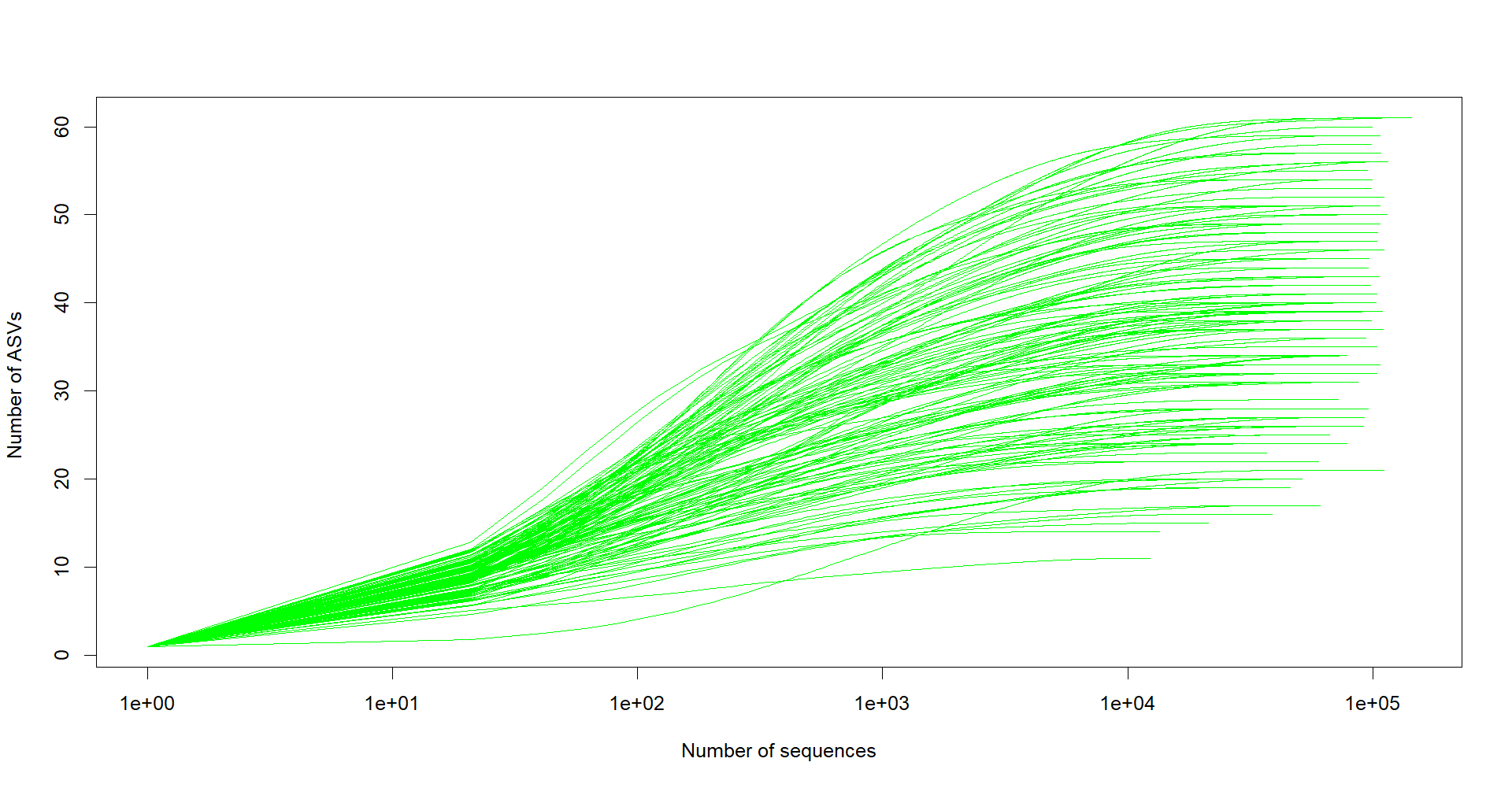
Appendix 1: Rarefaction curves**

**
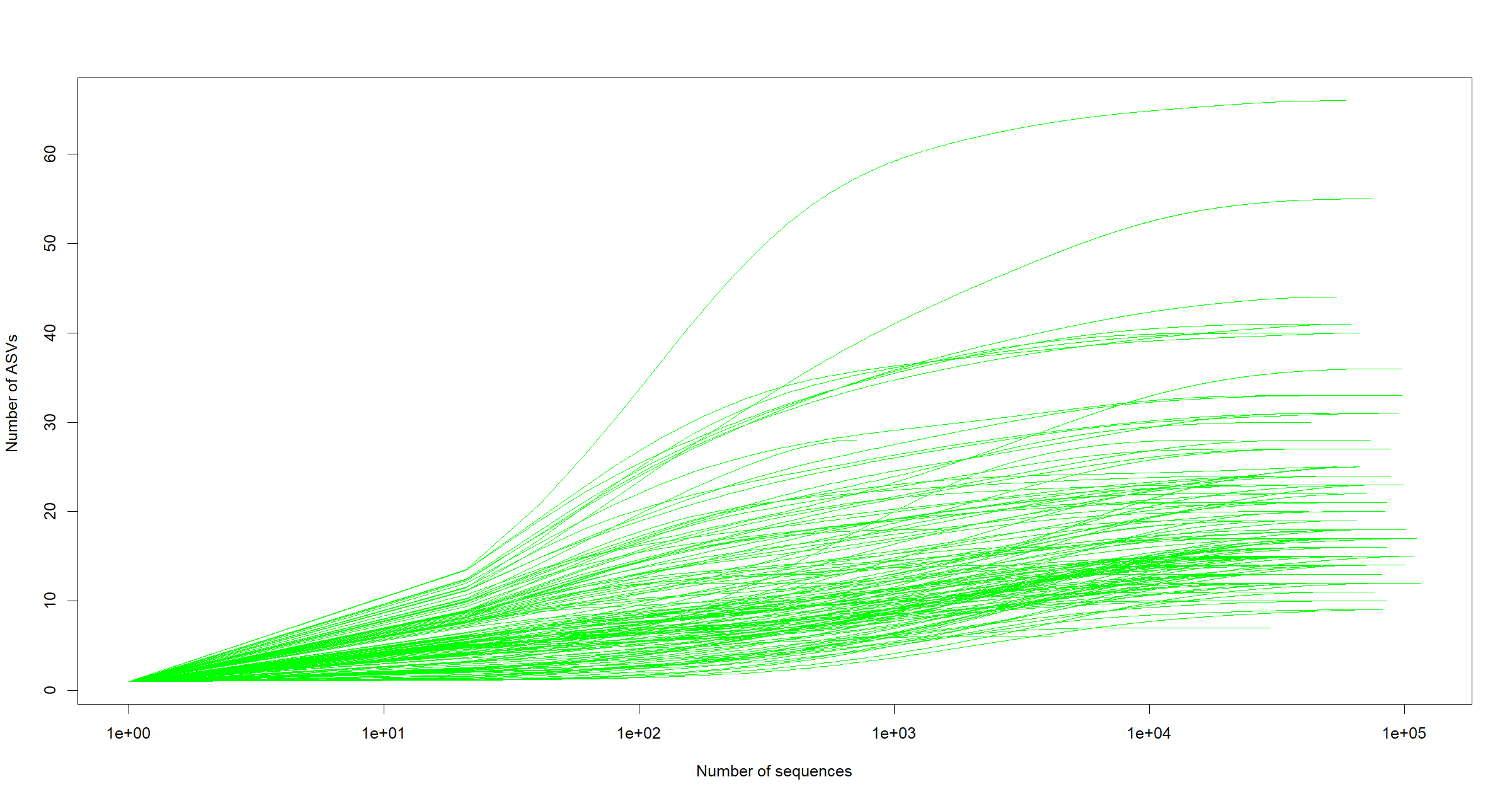
Fig S1. Rarefaction curves of sheep fecal samples, including all arthropod ASVs**. The rarefaction curves of most of the samples reached an asymptote level at around 10,000 sequences, suggesting that our sampling effort was sufficient to obtain a full estimate of ASV richness. Accordingly, the data was rarefied at 10,000 reads per sample.

**Fig S2. Rarefaction curves of cattle fecal samples, including all arthropod ASVs**. The rarefaction curves of most of the samples reached an asymptote level at around 10,000 sequences, suggesting that our sampling effort was sufficient to obtain a full estimate of ASV richness. Accordingly, the data was rarefied at 10,000 reads per sample.

**
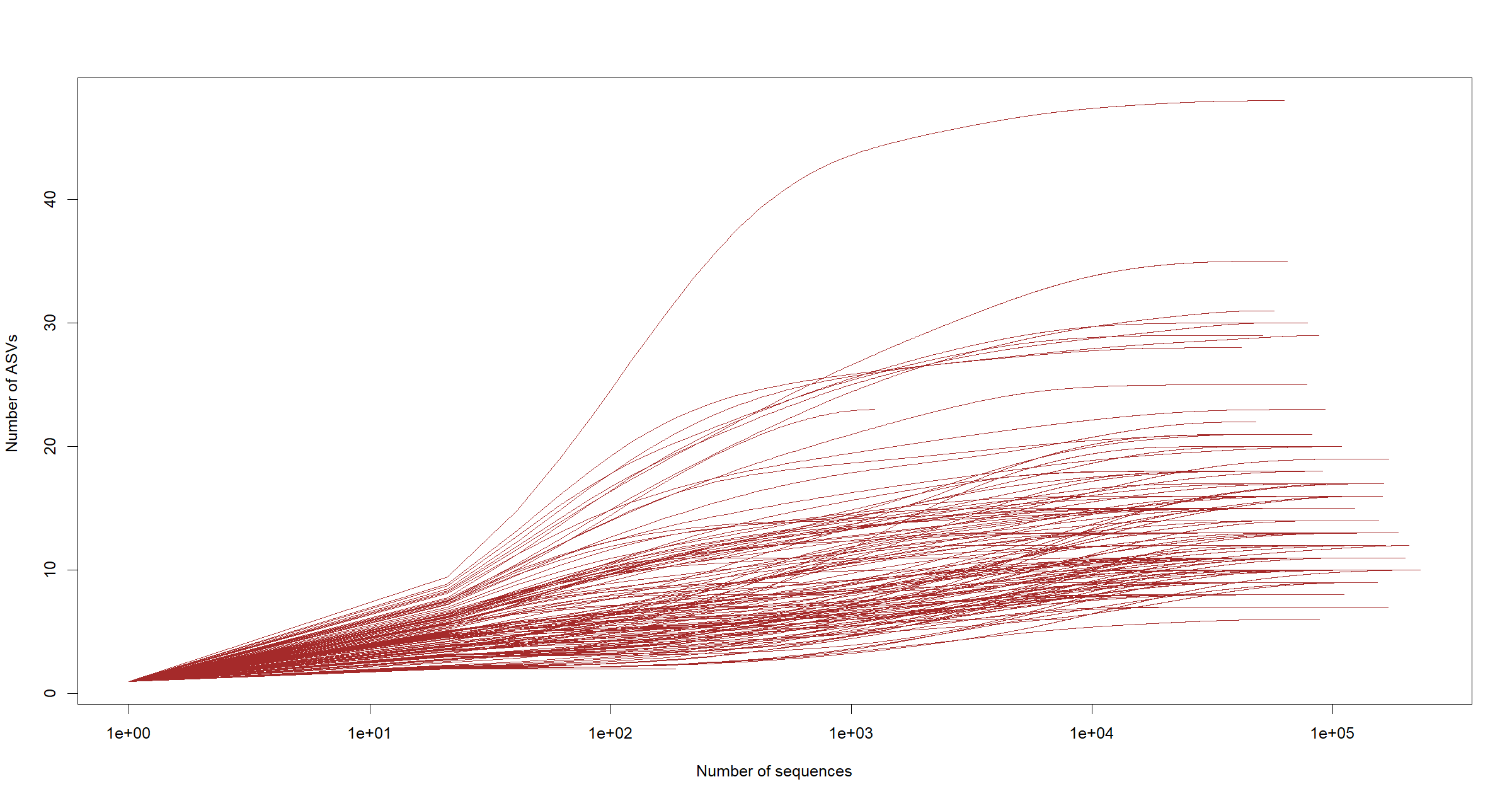

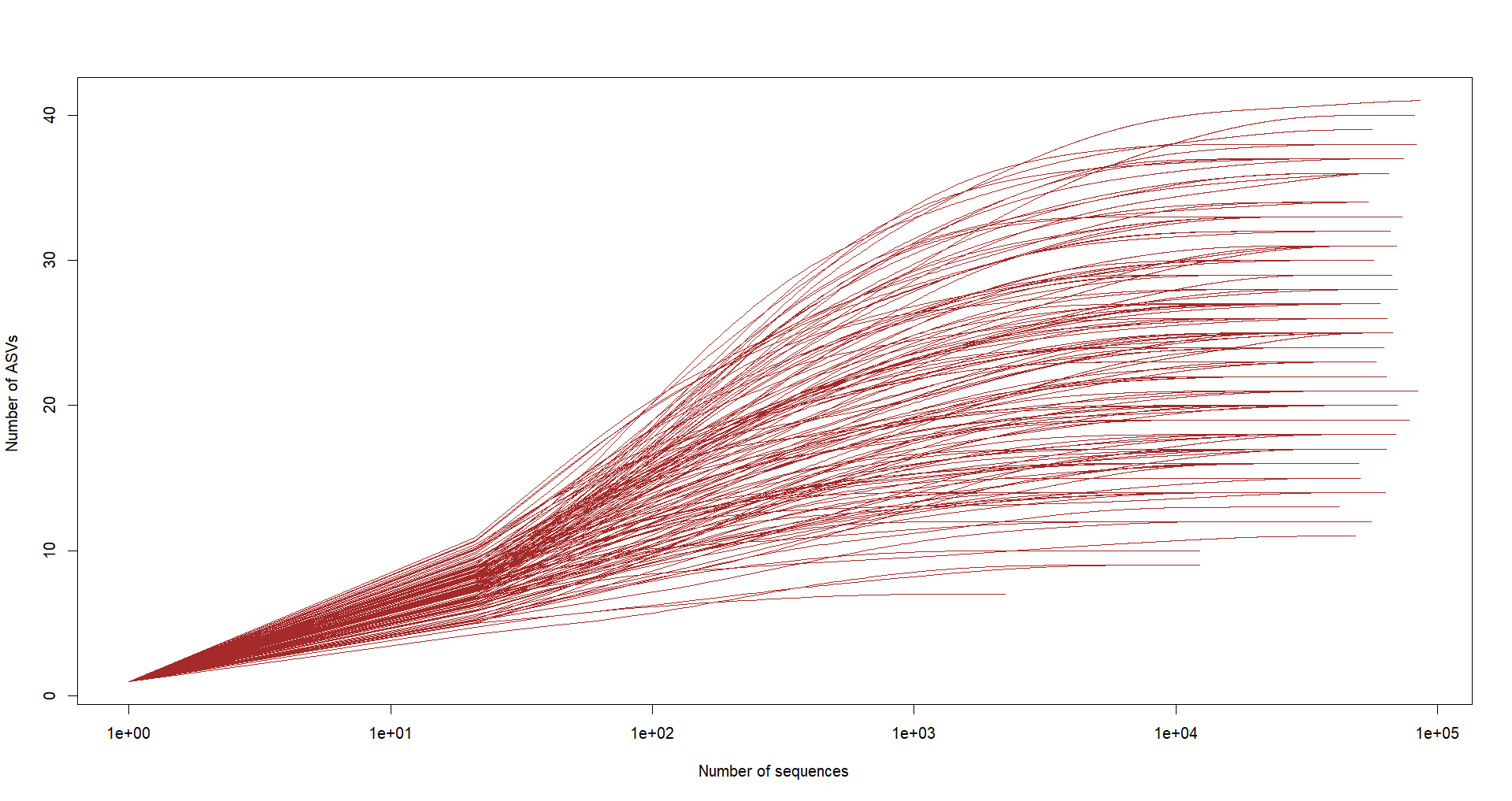
Fig S3. Rarefaction curves of sheep fecal samples including ASVs belonging to plant-dwelling arthropods (PDA)**. The rarefaction curves of most of the samples reached an asymptote level at around 10,000 sequences, suggesting that our sampling effort was sufficient to obtain a full estimate of ASV richness. Accordingly, the data was rarefied at 10,000 reads per sample.

**Fig S4. Rarefaction curves of cattle fecal samples including ASVs belonging to plant-dwelling arthropods (PDA) ASVs**. The rarefaction curves of most of the samples reached an asymptote level at around 10,000 sequences, suggesting that our sampling effort was sufficient to obtain a full estimate of ASV richness. Accordingly, the data was rarefied at 10,000 reads per sample.
