# Supplementary material for: Incidental Ingestion of Plant‐Dwelling Arthropods by Sheep and Cattle in the Same Habitat
Source: Ecol Evol. 2025 Jul 2;15(7):e71681. doi: 10.1002/ece3.71681 (PMC12222620; doi:10.1002/ece3.71681)
Supplement: Supplementary file 2 — Appendix S2. [file ECE3-15-e71681-s002.docx]

**Incidental ingestion of plant-dwelling arthropods by sheep and cattle in the same habitat**

**Appendix 2: Percentage of occurrence (POO) data**

This study utilizes the relative read abundance (RRA) approach to present its key findings, as this method can provide a relatively accurate view of population level estimates. Yet, RRA is sensitive to recovery biases and might under or overestimate the actual amount of a food item present if the efficiency of extracting DNA from different food sources varies (Deagle et al., 2018). Conversely, using only presence/absence data is considered a more conservative approach, but it can introduce biases like overestimating the importance of less common dietary components. To capitalize on the strengths of both methods, we conducted a complementary analysis using presence/absence data expressed as percentage of occurrence (POO).

We found consistent patterns between the RRA and POO data in both sheep and cattle datasets, across both the total arthropods and PDA. To confirm these similarities, we performed Mantel tests, which assess the correlation between distance matrices, comparing RRA- and POO-based distance matrices for both the full arthropod and PDA datasets in sheep and cattle fecal samples. In all four tests we found a strong positive correlation (Table A1), indicating a close similarity between the two methods. Therefore, the strong correlations observed between RRA and POO data support the reliability of the RRA-based conclusions presented in the main text.

**Table A1:** **Mantel test results for the correlation between RRA- and POO-based distance matrices for all arthropods and for plant-dwelling arthropods (PDA) alone, in sheep and cattle fecal samples.**

|  | All arthropods | | PDA only | |
| --- | --- | --- | --- | --- |
|  | P value | Correlation coefficient (r) | P value | Correlation coefficient (r) |
| Sheep | 0.001 | 0.92 | 0.001 | 0.9 |
| Cattle | 0.001 | 0.77 | 0.001 | 0.72 |
